# Distinct neural signatures of cognitive subtypes of dyslexia with and without phonological deficits^☆^

**DOI:** 10.1016/j.nicl.2013.03.010

**Published:** 2013-03-25

**Authors:** Muna van Ermingen-Marbach, Marion Grande, Julia Pape-Neumann, Katharina Sass, Stefan Heim

**Affiliations:** a Section Structural-Functional Brain Mapping, Department of Psychiatry, Psychotherapy and Psychosomatics, Medical School, RWTH Aachen University, Germany; b Section Neurological Cognition Research, Department of Neurology, Medical School, RWTH Aachen University, Germany; c Department of Psychiatry, Psychotherapy and Psychosomatics, Medical School, RWTH Aachen University, Germany; d Research Centre Jülich, Institute of Neuroscience and Medicine (INM-1), Germany; e JARA-Translational Brain Medicine, Germany

**Keywords:** Reading, Neuroimaging, Children, Dyslexia, Phonological deficit, Cognitive subtypes

## Abstract

Developmental dyslexia can be distinguished as different cognitive subtypes with and without phonological deficits. However, despite some general agreement on the neurobiological basis of dyslexia, the neurofunctional mechanisms underlying these cognitive subtypes remain to be identified. The present BOLD fMRI study thus aimed at investigating by which distinct and/or shared neural activation patterns dyslexia subtypes are characterized. German dyslexic fourth graders with and without deficits in phonological awareness and age-matched normal readers performed a phonological decision task: does the auditory word contain the phoneme/a/? Both dyslexic subtypes showed increased activation in the right cerebellum (Lobule IV) compared to controls. Subtype-specific increased activation was systematically found for the phonological dyslexics as compared to those without this deficit and controls in the left inferior frontal gyrus (area 44: phonological segmentation), the left SMA (area 6), the left precentral gyrus (area 6) and the right insula. Non-phonological dyslexics revealed subtype-specific increased activation in the left supramarginal gyrus (area PFcm; phonological storage) and angular gyrus (area PGp). The study thus provides the first direct evidence for the neurobiological grounding of dyslexia subtypes. Moreover, the data contribute to a better understanding of the frequently encountered heterogeneous neuroimaging results in the field of dyslexia.

## Highlights

• Dyslexia subtypes have a neurophysiological repercussion.
• Dyslexia subtypes are associated with distinct left fronto-parietal brain activation.
• All dyslexics vs. controls show enhanced activation in right cerebellum.

## 1 Introduction

The present paper investigates the neurobiological underpinnings of reading problems in developmental dyslexia. In particular, the fMRI study reported here sought to elucidate the neurobiological differences between different subtypes of dyslexic children with vs. without additional deficits in phonological awareness and also to assess commonalities between dyslexia subtypes. Consequently, we first give an introduction to cognitive models of dyslexia and recent findings on dyslexia subtypes. Next, we review the literature on the overall neurobiological foundation of dyslexia, with a particular focus on the heterogeneity of findings. It will be argued that this heterogeneity may in fact be the consequence of mixing different dyslexia subtypes in neuroimaging studies and of using paradigms which are more or less directly related to the deficits of these subtypes. This, in turn, implies the necessity of directly comparing brain activation patterns in dyslexic children with distinct, diagnostically clearly defined deficits.

### 1.1 Cognitive models of reading and dyslexia

Within the past decades, a variety of theories have been proposed in order to explain the diversity of linguistic and cognitive symptoms observed in developmental dyslexia. These theories conceptualize dyslexia as related to deficits that are either *phonological* (Liberman, 1973; Snowling, 1995, 2000; Vellutino and Fletcher, 2005; Vellutino et al., 2004), *attentional* (Bosse et al., 2007; Buchholz and Davies, 2005; Dubois et al., 2010; Facoetti and Molteni, 2001; Facoetti et al., 2001, 2003; Hari and Renvall, 2001; Pammer et al., 2004; Peyrin et al., 2008, 2011; Valdois et al., 2003, 2009), *visual-magnocellular* (Livingstone et al., 1991; Lovegrove et al., 1986; Stein, 1991, 2001, 2003; Stein and Fowler, 1993; Stein and Walsh, 1997; Vidyasagar, 2004), *auditory* (Tallal, 1980), or *automaticity*/*procedural learning* (Nicolson and Fawcett., 2005; Nicolson et al., 1991, 2001).

The most influential theory is the *phonological deficit hypothesis*, which offers a detailed explanation of reading and writing difficulties caused by language deficiencies within the phonological domain (Shaywitz and Shaywitz, 2005; Snowling, 1995, 2000; Vellutino and Fletcher, 2005; Vellutino et al., 2004). According to this theory, dyslexic readers have poor phonological representations, which in turn lead to failure in the ability to segment phonemes and is manifested in the specific difficulty of reading pseudowords correctly. This approach is not undisputed. Criticism comes from two sides. First, the phonological account of dyslexia is principally based on findings from English, a language with an irregular orthography. However, children learning regular orthographies like that of German have little difficulty with independent word recognition via phonological recoding in contrast to learners of irregular orthographies (Landerl et al., 1997a,b; Wimmer and Goswami, 1994; Ziegler et al., 2001), but they suffer from a pervasive speed deficit for all types of reading tasks (Wimmer, 1993). Second, other processing deficits (e.g. procedural learning or auditory discrimination abilities) are not only found repeatedly in dyslexia, but are also considered as underlying, (i.e. causing phonological deficit). Finally, these other processing deficits may even occur in the absence of phonological problems.

In general, there is increasing evidence that, in fact, different cognitive deficits in dyslexia may occur not only in concert, but also independently of one another (Bosse et al., 2007; Heim et al., 2008, 2010a,b; Ramus et al., 2003; Valdois et al., 2003; cf. Heim and Grande, 2012). Heim et al. (2008) classified German dyslexic children into different groups with distinct cognitive patterns. The existence of subtypes within the dyslexic sample was detected by a two-step cluster analysis, which identified three clusters of dyslexics with either a pure phonological deficit, an attentional deficit, or a combined deficit in phonological, attentional and magnocellular demands. Thus, Heim et al. (2008) demonstrated the existence of dyslexics without phonological deficits in a German dyslexic sample.

### 1.2 A neurocognitive basis for dyslexia subtypes?

While several cognitive subtypes are differentiable by their behavioral performances, one could assume that they are also differentiable by their neurofunctional activation patterns. Therefore, it is essential to detect neurocognitive differences between dyslexics with and without phonological deficits to obtain a more detailed understanding of the neurobiological basis of dyslexia. The actual state of research on the neurocognitive basis of dyslexia will now be reported in more detail.

#### 1.2.1 The neurobiology of dyslexia: Anatomy

In the literature, there are many neuroimaging studies on the structural and functional particularities of dyslexia. Both neuropathological data (Galaburda et al., 1985, 1994; Jenner et al., 1999; Livingstone et al., 1991; for a review see Wajuihian, 2011) and Magnetic Resonance Imaging (Eckert, 2004; Eckert et al., 2003; Larsen et al., 1990; for a review see Richlan et al., 2012) have yielded heterogeneous findings concerning structural differences in the brains of dyslexics and normally reading individuals. While some studies identified structural differences in the right inferior frontal gyrus, the left temporal-parietal region in bilateral medial occipital lobe (lingual gyrus) and in the anterior and posterior lobes of the right cerebellum (Brown et al., 2001; Eckert et al., 2003; Robichon et al., 2000; for a review see Eckert, 2004; Habib, 2000), a meta-analysis of nine structural MRI studies of dyslexia (Richlan et al., 2012) only found bilateral differences in the superior temporal gyri.

#### 1.2.2 The neurobiology of dyslexia: Function

In addition to these studies of structural brain differences there are several studies examining functional activation differences between dyslexics and normal readers during different phonological tasks, with the direction of effect (hypo- vs. hyperactivation) varying among studies. Independent of the first language, dyslexics have shown *reduced activation* for visually presented stimuli in left posterior inferior temporal and middle occipital regions including the left fusiform gyrus (visual word form area) (Cohen et al., 2000, 2004; Devlin et al., 2006; Price and Devlin, 2003). However, differences in activation profiles in the inferior temporal junction have not consistently been found in transparent writing systems. For a German sample Wimmer et al. (2010) showed instead reduced activation for dyslexics compared to controls in the left ventral occipitotemporal (OT) region, in the inferior temporal gyrus and in the inferior frontal gyrus. For English-speaking dyslexics, decreased activation for phonological processing was also found in the left temporo-parietal cortex (Cao et al., 2006; Hoeft et al., 2006; Temple et al., 2001), which supports the mapping of phonology onto orthography, and in the left inferior frontal gyrus (Cao et al., 2006; Georgiewa et al., 1999; Richlan et al., 2009, 2011), which is involved in articulation and naming.

Compared to controls, *increased activation* in dyslexia has been observed in some studies, most frequently in the left inferior frontal gyrus (Georgiewa et al., 2002; Heim et al., 2010b; Hoeft et al., 2007; Richards et al., 2002; Salmelin et al., 1996; Shaywitz et al., 2004), whereas others have found no differential activations in left frontal regions (Rumsey et al., 1997; Temple et al., 2001). Although the inferior frontal gyrus seems relevant for both reading and phonological awareness, a consistent account is still lacking. The first steps towards an integrative approach relating cognitive demands and brain function were taken in two recent neuroimaging studies (Heim et al., 2010a,b). One study (Heim et al., 2010a) focused on the influence of reading proficiency on brain activation during phonological awareness and reorientation of attention predominantly in distinguishable patterns in left (phonological) versus right (attention shifting and visual motion detection) inferior frontal gyrus. Distinct left versus right frontal effects indicate different neural mechanisms and verify that phonological processing deficits in dyslexia do not necessarily result from impaired magnocellular functioning. The other study (Heim et al., 2010b) followed the reverse logic, investigating how brain activation during reading was modulated by the individual subject's level of phonological awareness as well as other cognitive variables. Here, hemispheric differences between dyslexics and normal readers were observed, with predominantly right-lateralized effects for co-variation effects in middle frontal gyrus, inferior parietal cortex, and precuneus in normal readers, but left-lateralized effects in corresponding regions in dyslexics.

### 1.3 Study aim

In summary, studies investigating phonology in dyslexic readers provide novel insights into the neurobiological mechanisms underlying dyslexia in general, which differ from those in normal readers. Yet, a direct comparison of brain activation during phonological tasks among sets of dyslexic children with and without phonological deficits and normal readers is missing. The present study aims to provide a more detailed look at phonological processing in cognitive subtypes of dyslexia from a neurofunctional perspective. The inconsistent findings in previous studies, with respect to hypo- or hyperactivation in different brain areas, might be explainable by the fact that these studies did not consider the existence of cognitive dyslexia subtypes. Rather, the described effect may have been borne by just one cognitive subtype which was over-represented in the study sample. We hypothesize that activation profiles for a phonological task differ not only between dyslexics and controls, but also between the two dyslexia subtypes. We would expect that dyslexics, in contrast to controls, would show increased activation profiles in left inferior frontal regions involving Brodmann area 44, given its previously reported role in phonological processing. Furthermore, we would expect subtype-specific differences in brain activation. Phonological dyslexics may reveal increased activation profiles in phonology-related areas while dyslexics without phonological deficits would not show hyperactivation. To attain an integrative approach to brain function and behavior, the present study correlates test scores of phonological processing and reading ability with activation intensity in brain areas differing between dyslexics and controls and between the two dyslexia subtypes. We expect that hyperactivation in dyslexics will be associated with lower test scores in phonological awareness and reading ability. For task performance inside the scanner, we hypothesize that controls will pass with distinction and that dyslexics with phonological deficits will perform significantly below the level of non-phonological dyslexics as well as controls.

## 2 Materials and methods

### 2.1 Participants

All procedures were designed in accordance with the Declaration of Helsinki and were approved by the ethics committee of the Medical Faculty of RWTH Aachen University (Reference number EK 153/08).

785 German primary school children (grades 3 and 4) were screened for participation in the study. Reading ability was assessed with the standardized reading screening SLS 1–4 (Salzburger Lesescreening SLS; Mayringer and Wimmer, 2003). The reading screening measured reading speed and basic reading ability (automaticity, accuracy) and is practicable as a group test. Children were asked to read as many sentences as possible within a time limit of 3 min and to mark each sentence as semantically correct or incorrect. The test manual reports high reliability (parallel test method; r = 0.90) and adequate validity (external criterion: r = 0.75). Among those children initially screened, 245 children showed deficient reading ability and 107 monolingual German-speaking children were further tested. Children with reading deficits (SLS Reading Quotient < 90), average intelligence (IQ > 85), and without auditory or visual perception deficits were defined as dyslexics. Children without reading deficits (SLS Reading Quotient ≥ 90), average intelligence (IQ > 85) and without auditory or visual perception deficits were defined as controls. 31 children with dyslexia (mean age = 9.9 years [range: 8.8–11.2; SD = .68]; 16 female) and 13 normal reading children (mean age = 9.6 years [range: 9.0–10.5; SD = .47]; 5 female) agreed to undergo fMRI scanning and were included in the study. Additional written informed consent was obtained from their parents. Based on their performance in a test of phonological awareness (see below), the dyslexic group was further divided into 17 children with and 14 children without phonological deficits.

### 2.2 Procedure

The standardized psychometric tests and paradigms employed for the assessment of cognitive function assessed non-verbal intelligence, visual and auditory perception, phonological awareness, reading, and writing, and will now be explained in more detail.

#### 2.2.1 Non-verbal intelligence

Non-verbal IQ was assessed with the German version of the *Cattell Culture Fair Test* (CFT 20; Weiß, 1998). Children were included in the study with an age-related IQ > 80 in order to exclude children with general learning disabilities.

#### 2.2.2 Visual and auditory processing

In order to exclude perceptual impairments that might influence reading ability, the pre-test of auditory and visual perception of the *Wiener Testsystem* (WAF, Häußler and Sturm, 2009), suitable at the age of eight years onwards, was administered. In this test battery, children performed discrimination tasks along the dimensions of brightness, shape and sound volume. Children with a value below 80 were excluded from the study.

#### 2.2.3 Phonological processing

The ability to segregate and manipulate phonemes from auditorily presented words (phonological awareness) was tested by the standardized German *Basiskompetenzen für Lese-Rechtschreibleistung* (BAKO 1-4; Stock et al., 2003) consisting of seven subtests: segmentation of pseudo-words, vocal substitution, phoneme categorization, phoneme commutation, word reversal, word identification after dropping the first phoneme and discrimination of vowel length. Separate norms are available for grades one to four.

#### 2.2.4 Visual scanning

The ability to systematically scan the surroundings for relevant information and consciously control attentional focus was tested as an additional psychometric assessment with the *Test of Attentional Performance for Children* (KiTAP; Zimmermann et al., 2002).

### 2.3 Dyslexic subgroups

Depending on the results of the phonological awareness test, each of the 31 dyslexic children was assigned to one of two dyslexia subgroups. T-values illustrate the relationship between a single outcome and a reference group with a median of 50 and standard deviation of 10. T-values under 40 are thus below average. Children with BAKO T-values ≤ 40 were classified as phonological dyslexics (N = 17; mean age 9.9 years (range: 8.8–11.2; SD = .79); 8 girls) and those with T-values > 40 were classified as non-phonological dyslexics (N = 14; mean age: 9.8 years (range: 8.7–10.11; SD = .55); 8 girls). The control group (N = 13, mean age: 9.6 years (range: 9.0–10.5; SD = .47); 5 girls) consisted only of children with good reading skills and BAKO T-values above 40.

### 2.4 fMRI paradigm

Brain activation for phonological processing was assessed in a phonological task. Instead of a resting baseline an auditory control task was conducted to reveal only brain regions involved specifically in phonological processing. The total experiment had a duration of 9 min and was created and analyzed as a block design. At the beginning of each block, instructions were presented visually for 3900 ms (phonological task: “A–no A”; control task: “left–right”), followed by a blank screen of 100 ms. Before each block, a random jitter varied the start of the sampling of brain volume images relative to the start of the stimulus presentation (0.25–1.00 s) to obtain a better sampling of the hemodynamic response. Before the presentation of the stimulus, a fixation cross was presented in the middle of the screen for 0.50 s. The stimulus itself had a duration of less than 1 s. After the stimulus, an empty screen was shown for 1 s, during which the children responded. There was a total of 16 blocks, eight for each condition, with a duration of 32 s (i.e. the duration of eight trials) each. The order of the blocks was not intermixed and always started with the control condition followed by the phonological task. The stimuli consisted of 64 pseudowords (previously used by Sass et al., 2009), which were randomized and presented via headphones randomly on the left or right channel for both conditions. In the phonological blocks, the children listened to the presented stimuli and decided whether they contained the phoneme/a/or not by pressing one of two response buttons with their left or right index finger, respectively. In the control task, participants had to decide if the stimuli were administered to the left or right ear by pressing the corresponding button. The set of language stimuli was identical for the phonological and the control task in order to exclude stimulus-related effects.

#### 2.4.1 Localization of effects using cytoarchitectonic maps

Cytoarchitectonic probability maps (Amunts et al., 2004) for the anatomical localization of the effects were obtained on the basis of mapping areas in histological sections using an observer-independent approach for the definition of cortical borders (Schleicher et al., 2005; Zilles et al., 2002). The cytoarchitectonic probability maps provide information about the position and variability of cortical regions within standard MNI reference space. The SPM Anatomy Toolbox (Eickhoff et al., 2005) facilitates the assignment of the MNI coordinates to cytoarchitectonically defined regions and is receivable with all cytoarchitectonic probability maps and references from http://www.fz-juelich.de/SharedDocs/Downloads/INM/INM-1/DE/Toolbox/Toolbox_18.html.

#### 2.4.2 MRI data acquisition and analysis

The fMRI experiment was carried out on a 3T Siemens Trio scanner (Siemens, Erlangen, Germany) at the University Hospital Aachen. A standard birdcage head coil was used and soft foam padding was utilized to reduce head motion. Echo-planar images (repetition time (TR): 4000 ms; field of view (FoV) 200 mm; flip angle 90°; and echo time (TE): 30 ms) were acquired from 40 transverse slices covering the entire brain (in-plane resolution: 3 × 3 mm^2^; slice thickness: 3 mm; and inter-slice gap: 1 mm).

In addition, anatomical images (orientation: sagittal; slice thickness: 1 mm; FoV = 250 mm, TR = 1900 ms, TE = 2.52 ms) were also obtained. Functional scans were analyzed with Statistic Parametric Mapping *SPM5* (Wellcome Department of Cognitive Neurology, London) and *Matlab 7* (The Mathworks Inc., Natick, USA). Pre-processing involved the standard procedure of realignment to the first image, slice timing, normalization to standard MNI space,^1^ and smoothing with a Gaussian kernel of 8 mm FWHM. All images were motion controlled with a median of x = .0 (range: − .45 mm to .07 mm), y = .12 (range: − .74 mm to 1.54 mm); z = − .08 (range: − 1.62 mm to 1.23 mm); pitch = 0.0 (range: − .05° to .02°); roll = 0.0 (range: − .01° to .04°); yaw = 0.0 (range: − .01° to .06°).

At the first level, a within-subject analysis was performed where activation was averaged across scans for every individual subject for the phonological and control task separately. These contrast images were then entered into the second-level random effect group analysis. The second-level analysis was performed using a flexible factorial design for repeated measures, allowing the assessment of main effects for differences between the phonological and the control task as a function of Group. Taking the contrast of the phonological task against the control task (Ptask > Ctask) as the basis for the following analyses, the interaction term of group and task reveals the brain regions that differentiate the three groups with respect to phonological processing independent of auditory processing. The contrast between the phonological task and the control task was analyzed using a one-way ANOVA for the whole sample and for each group separately (Fig. 2; Appendix Table A.1). The following contrasts were computed at the second level with IQ as a covariate of no interest. A flexible factorial model with the factors “subject”, “group” and “condition” was used. To show activation differences between both subtypes compared to controls, a conjunction analysis composed of phonological dyslexics versus controls and non-phonological dyslexics versus controls was performed (PhoDys > Con ∩ NonPhoDys > Con). This contrast allows a direct comparison of the results to the existing literature. The single contrasts of the dyslexic subgroups versus controls are displayed in the Appendix A (Appendix Table A.2). To show activation differences between the two dyslexic subgroups, we contrasted the phonological dyslexics versus the non-phonological dyslexics and masked this contrast inclusively with the contrast of the phonological dyslexics versus the controls. This contrast thus shows the phonological activation effect which is specific for the phonological dyslexics. The reverse contrast in regard to the specific effect of the non-phonological dyslexics was also computed.

To visualize the differences in phonological awareness and reading ability as a unique identifier for group and to show the dependency between the scores of phonological awareness and reading ability, a correlation analysis between these two cognitive demands was performed.

## 3 Results

### 3.1 Behavioral data

For an overview, the behavioral data from the diagnostic session before scanning are shown in Table 1 and were further analyzed with *SPSS* for Windows version 19.0 (SPSS, Inc., Chicago, Illinois). The reading screening showed significant differences between the groups (F_2,41_ = 59,285; p < .001). The Scheffé test for multiple comparisons indicated that the phonological dyslexics and the non-phonological dyslexics did not differ significantly from each other (p = .998), but both dyslexic groups differed significantly from the controls (p < .001). To test for a dissociation between the two dyslexic subgroups in phonological awareness, a one-way ANOVA with *SPSS* 19.0 was performed. The comparison of the mean T-values of the phonological awareness test of all three groups showed significant differences (F_2,41_ = 35.38; p < .001). Even the pair-wise comparison of the groups revealed significant differences in phonological awareness (phonological dyslexics (PhoDys) and controls (Con): p < .001; PhoDys and non-phonological dyslexics (NonPhoDys): p < .001; and NonPhoDys and Con: p < .004).

**Fig. 1:**
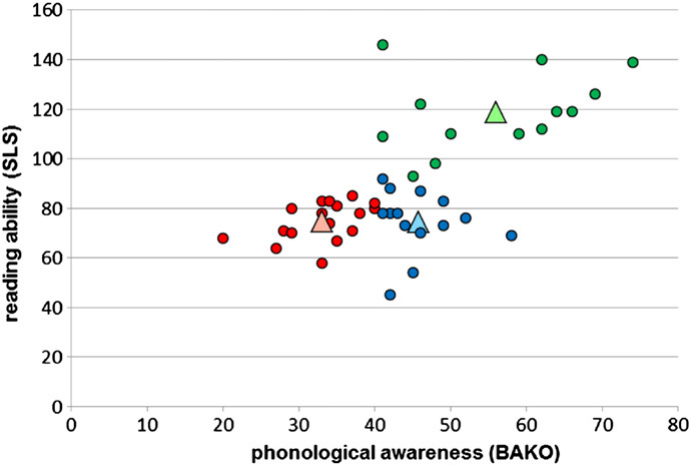
Assignment of the participants to the phonological dyslexic group and the non-phonological dyslexic group on the basis of the results of the phonological awareness test (BAKO) and reading quotient (SLS). Notes: Phonological dyslexics are shown in red with BAKO T-values < 40 and an SLS reading quotient below 90. Non-phonological dyslexics are shown in blue with BAKO T-values > 40 and an SLS reading quotient below 90. The controls are shown in green with BAKO T-values > 40 and an SLS reading quotient above 90. The large triangles show the mean value of each group.

To show the linkage of phonological awareness and reading quotient, a correlation analysis was performed. Considering both dyslexic subgroups as one big group, a strong correlation (r = .646; p < .001) between phonological awareness and reading ability emerged. The better the phonological awareness, the greater were the reading skills (Fig. 1). The analysis of IQ level also showed group differences (F_2,41_ = 5812; p = .006). Again, the Scheffé test did not yield differences between the phonological dyslexics and the non-phonological dyslexics (p = .827). The controls showed significantly higher IQ level than the phonological (p = .008) and non-phonological dyslexics (p = .048).^2^

The behavioral data for all participants during scanning, i.e. represent the percentage of correct key presses and reaction time, are shown in Table 2.

For all groups the analysis of the behavioral data showed significantly better performance on the control task than on the phonological task (F_1,41_ = 34.33; p < 0.001). There was no main effect for group and task separately, but the interaction of these variables was significant (F_2,41_ = 4.29; p = 0.02): The phonological dyslexics showed the biggest differences between correct responses on the control and the phonological tasks followed by controls and non-phonological dyslexics. The post-hoc Least Squares Difference (LSD) test for pair-wise group comparison showed that, concerning the interaction, the phonological dyslexics differed significantly from the non-phonological dyslexics (p = .006) but not from the controls (p = .463). The differences between the phonological and non-phonological dyslexics were slightly below significance (p = .055).

Next, we resolved the interaction term, analyzing the significance of the task difference (correct responses for the phonological minus the control task) among the three groups. In general, the differences between the phonological and the control task showed a significant main effect for Group (F_2,41_ = 4.29; p = 0.02). This main effect was driven by the significant difference between the phonological dyslexics and the non-phonological dyslexics. The post-hoc Scheffé test allows a group-wise comparison of the task differences described above. This comparison test showed that the phonological dyslexics had a significantly greater difference between the phonological and the control tasks than the non-phonological dyslexics (p = .025), with better performance on the control task. Task differences between the non-phonological dyslexics and controls were present but not significant (p = .154). Both groups showed better performances on the control task than on the phonological task. When comparing the phonological dyslexics with the controls in terms of task differences, the Scheffé test showed no significant effects (p = .762). Thus, the phonological dyslexics and the controls showed nearly the same differences between the phonological task and the control task.

Reaction times on the control task were significantly faster than on the phonological task (F_2,43_ = 113.96; p < .001) but no significant group difference was found.

In summary, the behavioral data from testing inside the scanner revealed significant differences between the phonological task and the control task among the dyslexic subgroups, whereby the phonological dyslexics performed worse on the phonological task compared to the non-phonological dyslexics and the controls. As to performance on the phonological task minus the control task, there were no significant differences between the non-phonological dyslexics and the controls. For the phonological task and the control task separately there were no significant differences between the three groups.

Lastly, we wanted to establish whether inside the scanner resembled that outside the scanner. Therefore, the performance in the standardized BAKO test of phonological awareness outside the scanner was correlated with the performance in the phonological task inside the scanner (controlling for initial reading score and response speed). This partial correlation was moderate (r = .258, p = .05).^3^

### 3.2 fMRI data

**Fig. 2:**
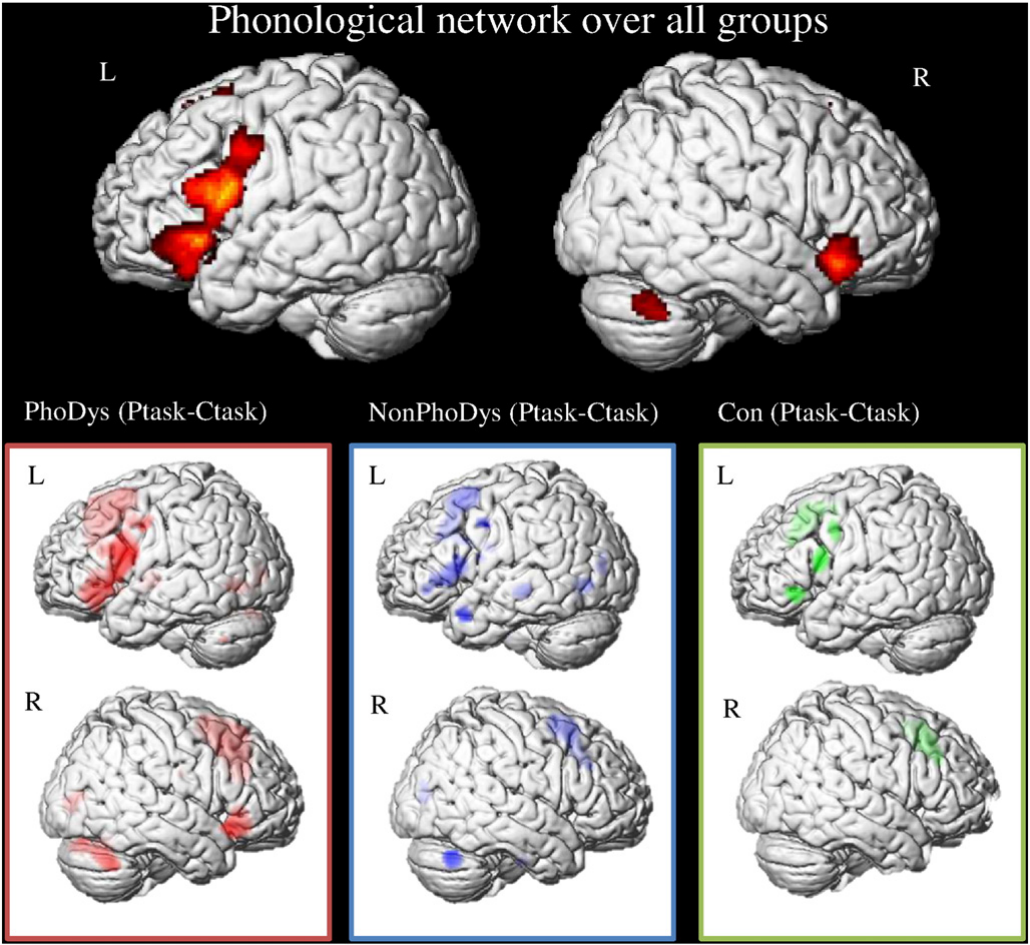
Brain activation differences between the phonological task (Ptask) and the control task (Ctask). The top image above shows the phonological network for the total sample. Each group is presented separately below: controls (Con; green), phonological dyslexics (PhoDys; red) and non-phonological dyslexics (NonPhoDys; blue), cluster size k ≥ 10 voxel (local maxima significant at p < .001 uncorrected).

To identify regions used for phonological processing in general, we contrasted the phonological task with the control task for all groups combined and for each group separately (p = .001, uncorrected, threshold k = 10). The contrast for the total sample revealed significant activation in bilateral insula (Ig2; Kurth et al., 2010a,b), left inferior frontal gyrus (IFG, areas 44,45; Amunts et al., 1999), supplementary motor area (SMA, area 6; Geyer, 2003), left lingual gyrus (areas 17,18; Amunts et al., 2000), right cerebellum (lobule VIIa Crus I (Hem), Lobule VI (Hem); Diedrichsen et al., 2009), left thalamus (Th-prefrontal), and left superior parietal lobe (hiP3; Scheperjans et al., 2008) (see Fig. 2; Appendix: Table A.1). All further group comparisons were based on the contrast of the phonological task versus the control task and will now be explained in more detail.

#### 3.2.1 Dyslexics versus controls

**Fig. 3:**
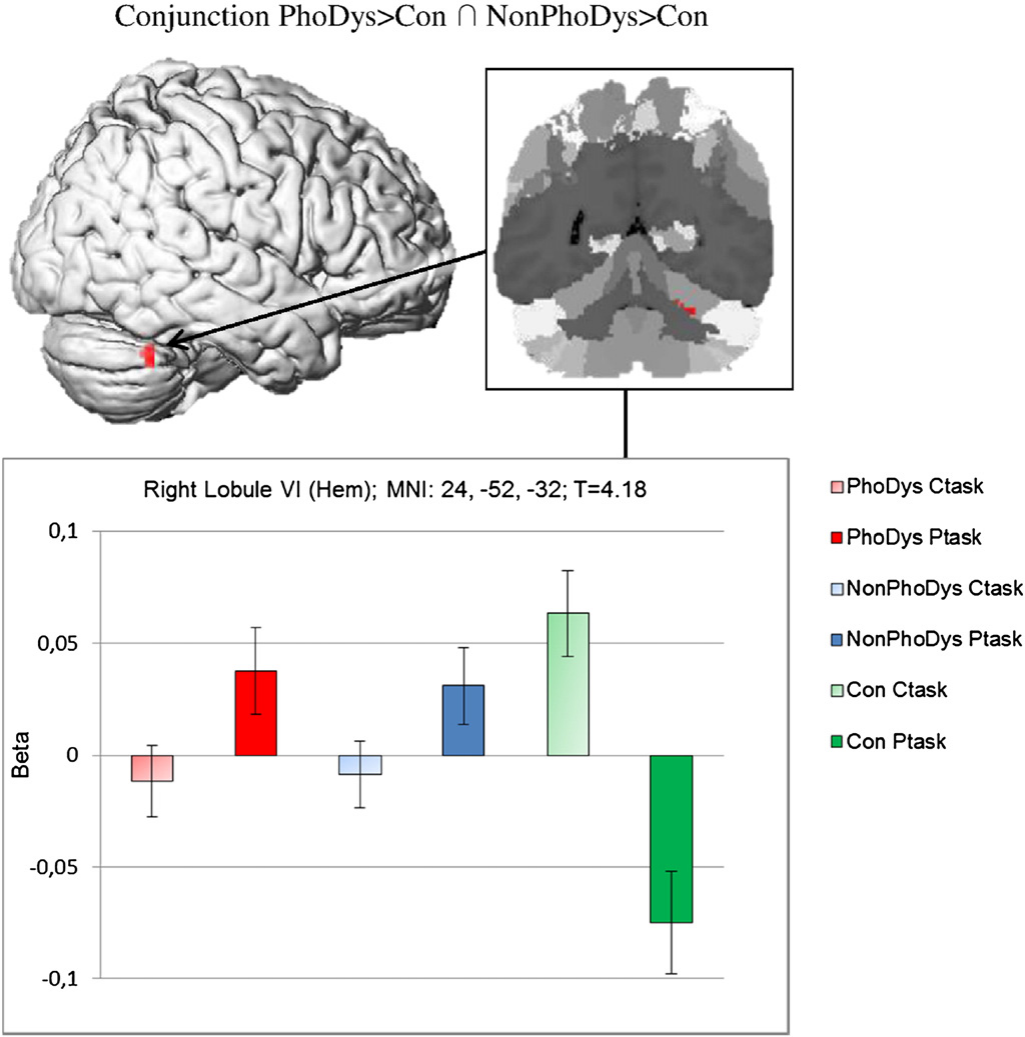
Conjunction analysis of phonological dyslexics versus controls and non-phonological dyslexics versus controls (PhoDys > Con ∩ NonPhoDys > Con), with a cluster size k ≥ 10 voxel (local maxima significant at p < .001 uncorrected). The bar graphs represent the activation power (Beta) of the phonological task minus the control task in the right cerebellum for phonological dyslexics (PhoDys; red); non-phonological dyslexics (NonPhoDys; blue) and controls (Con; green).

The conjunction analysis of the phonological dyslexics compared to controls and non-phonological dyslexics compared to controls revealed increased activation for both groups of dyslexics in the right cerebellum (Lobule VI (Hem)) performing a phonological task (p = .001, uncorrected, threshold k = 10; see Table 3; Fig. 3). There was no decreased activation found for the dyslexics compared to the controls. The present study did not focus on the direct comparison of each subtype to the controls separately. For completeness the results of the phonological dyslexics versus controls and non-phonological dyslexics versus controls are shown in the Appendix, Table A.2.

#### 3.2.2 Direct comparison of the two dyslexic subgroups

**Fig. 4:**
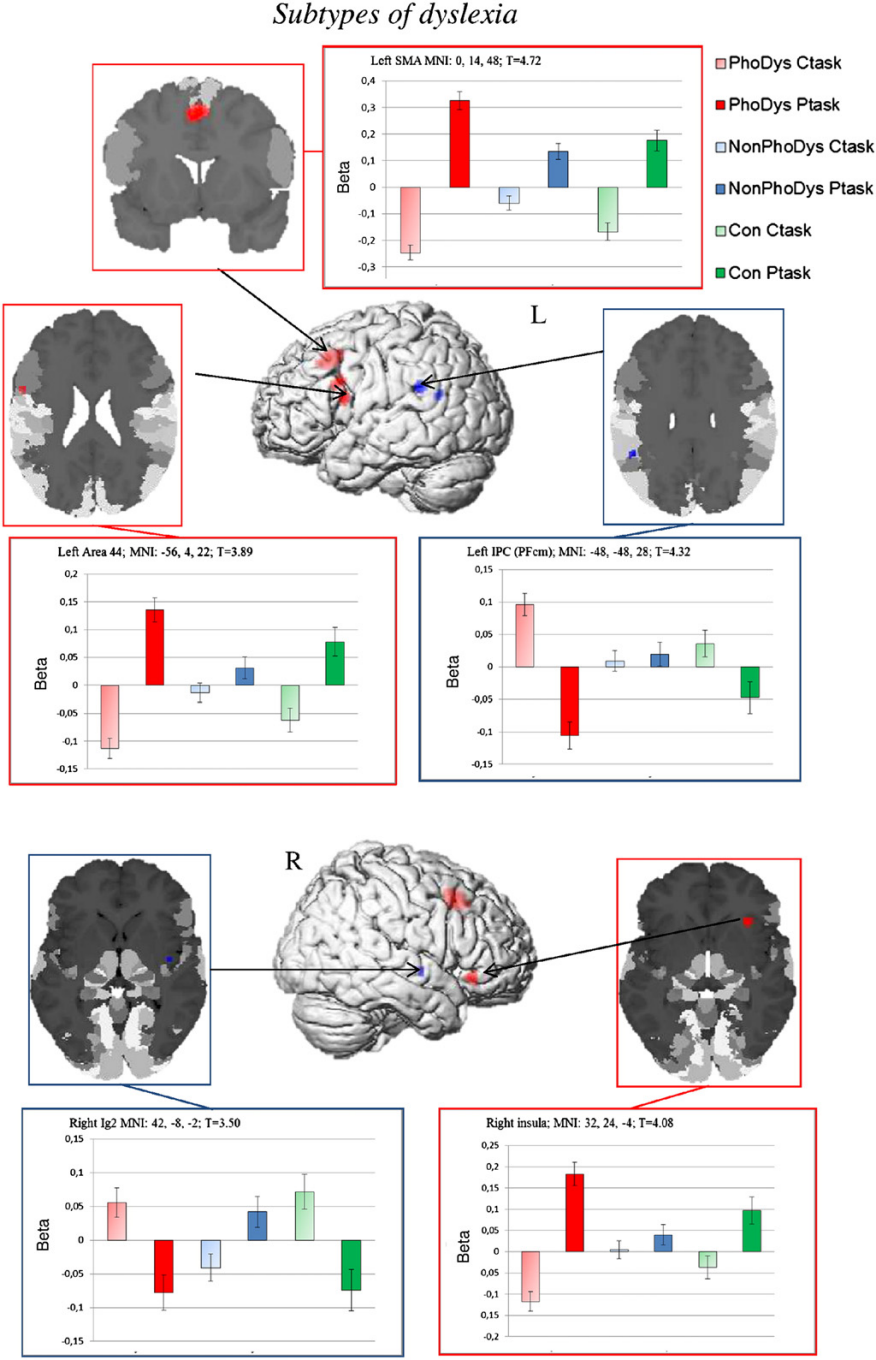
Subtype-specific brain activation profiles for phonological dyslexic (PhoDys; red) and non-phonological dyslexics (Non-PhoDys; blue). PhoDys compared to NonPhoDys (and reverse) were examined and masked inclusive for those regions that showed significant effects in PhoDys versus controls (Con; green). All clusters have an extent size of k ≥ 10 voxel and local maxima are significant at p < .001 uncorrected.

We determined the regions in which the phonological dyslexics exhibited higher activation in the phonological task compared to the non-phonological dyslexics. To identify the specific activation cluster for phonological dyslexics, this contrast was masked with the contrast of the phonological dyslexics versus controls (p = .001, uncorrected, extent threshold k = 10). The results showed that the activation profile of the phonological dyslexics looked more similar to that of the controls but with greater activation intensity (bar graph Fig. 4). The phonological dyslexics exhibited increased activation bilaterally in cytoarchitectonic area 6 in left SMA, left inferior frontal gyrus (area 44, with the peak of the cluster in the posteriorly adjacent precentral gyrus) and in the right insula (see Table 4 and Fig. 4) compared to non-phonological dyslexics.

The reverse contrast showed that the activation differences between the dyslexics without phonological deficits compared to the phonological dyslexics and controls were significant in the left supramarginal and angular gyrus (areas PFcm and PGp; Caspers et al., 2006) (Fig. 4; Table 4).^4^

## 4 Discussion

The present study has demonstrated that the neurofunctional organization of phonological processing differs in cognitive subtypes of dyslexic children with high vs. low phonological awareness. Despite the fact that children activated parts of the phonological network were activated in all children, there were characteristic differences between dyslexic and normally reading children. The study thus documents that cognitive subtypes of dyslexia are distinguishable not only with respect to their performance, but also with respect to their brain activation patterns as the putative neuronal basis of performance.

### 4.1 The phonological network

Contrasting the phonological task to the control task, every group yielded brain areas such as left area 44 (Burton, 2001; Démonet et al., 1992), the insular cortex (Damasio & Damasio,1983), the junction of the superior parietal lobule and the superior occipital gyrus (Seghier et al., 2004), area 6 (Seghier et al., 2004) and the cerebellum (which exerts a more indirect influence on phonological processing; Desmond and Fiez, 1998; Nicolson and Fawcett, 2005) all said to be involved in phonological processing. The contrast between the phonological and the control task served as the basis for all subsequent group analyses, which consisted of interactions between groups and tasks. The group comparisons aimed to show how brain regions are uniquely involved in the phonological component of the task and differ between groups which, overall, recruit parts of the phonological network. Despite these overall comparable patterns of activation, there were robust differences both between dyslexics and normal readers and, most importantly, between the two dyslexic subtypes. These differences are discussed in the following paragraphs.

### 4.2 Differences in phonological processing of dyslexics and controls

#### 4.2.1 Behavioral data

As mentioned in Section 3.1, the controls showed a significantly higher IQ level than the dyslexics, whereas the phonological and non-phonological dyslexics exhibited comparable IQ (but note the comparable results in an additional analysis with an IQ-matched sample, cf. Table 2). Because the present study aimed to show differences between the two dyslexics groups, this circumstance seems to be acceptable, whereas the impact of intelligence on phonological processing could not be proved in a previous study (Temple et al., 2001).

Classifying the dyslexic children into cognitively distinguishable subgroups (i.e., groups with and without a phonological deficit) revealed differences for both performance in the scanner and for brain activation patterns. The subtype of dyslexics without phonological deficits could possibly reflect the benefits of the greater transparency of the German orthography for beginning readers. Wimmer and Goswami (1994) mentioned that the transparency of orthography might affect the development of reading in a direct way due to the simplicity of grapheme-to-phoneme conversion and in an indirect way attributable to teaching method. German children have the benefit of a very transparent writing system, which could be manifested in fewer phonological deficits than in languages with irregular orthography and so a new subtype of dyslexia without phonological deficits may be observed more frequently in these languages. Wimmer et al. (1991) found that most of the German children had already overcome initial phonological difficulties by the end of the second grade in primary school. However, at the time point of the present experiment, the non-phonological dyslexics (be they early compensators of phonological problems or not) were clearly distinct from those dyslexics with a phonological deficit. To be sure that the non-phonological dyslexics did not overcome phonological deficits at an early stage the phonological ability of the children needs to be pretested in kindergarten.

With regard to potential dyslexic subtypes, the *Test of Attentional Performance for Children* (KiTAP; Zimmermann et al., 2002) was performed as an additional psychometric assessment. A T-test comparing mean values showed that the non-phonological dyslexics exhibited significantly lower scores in visual scanning than the healthy controls did (p < .032). It might be that the underlying cognitive deficit of the non-phonological dyslexics potentially lies in the attentional system, especially in controlling the direction of attentional focus. These results are in line with previous findings from Heim et al. (2008), who described a dyslexic subtype suffering from deficits in attentional shifting (Posner, 1980).

As expected, the analysis of the behavioral data of the phonological task conducted in the scanner indicated that the phonological dyslexics performed significantly worse than the non-phonological dyslexics and controls. Surprisingly, the non-phonological dyslexics performed even better than controls. This could be due to the fact that some of the dyslexics without phonological deficits had had continued speech intervention in the past, whereas none of the controls received such therapy. It might be that the dyslexics without phonological deficits had overcome potential phonological problems and had more training in this kind of task than the controls.

The auditory stimuli were presented on the right or left channel in a randomized order. To eliminate any possibility of negative effects on the results caused by retarded auditory pathway development, we analyzed the number of correct answers in the control task for each channel of stimulus presentation separately. This approach refers to the well-investigated right ear advantage (Hugdahl, 2011), even though our paradigm did not involve the classical dichotic listening situation. The right ear advantage, usually observed for dichotic listening, is the asymmetry of reports for the right ear versus the left ear characteristically for speech sounds (Hugdahl, 2011). Left and right ear correct scores were compared for ear advantage in each of the two dyslexic subgroups and controls. Over all groups, the differences between correct answers when the stimulus was presented to the left or right ear was slightly below significance (p = .051). Phonological (p = 1.0) and non-phonological dyslexics (p = .135) showed no preference for one ear, whereas the controls yielded a significant right ear advantage (p = .006). Previous studies have reported that healthy right-handed children on average exhibited a right ear advantage (Bryden, 1970; Kimura, 1963), whereas dyslexics showed an attenuation of the right ear advantage (Cohen et al., 1992; Hugdahl et al., 1995; Witelson and Rabinovitch, 1972). Thus, the alternate presentation of the stimuli in the present study cannot explain the poorer performance of the phonological dyslexics, because the absence of a right ear advantage was also shown in non-phonological dyslexics.

Reaction times on the control task were significantly faster than on the phonological task, which might indicate an enhanced cognitive effort on the phonological task. The performance of the children manifested itself in correctness and not in the reaction times which explains the absence of an interaction between group and reaction time.

These reaction time data were largely in line with the subjects' performance in a standardized test of phonological awareness. The correlation was positive, though moderate. Possible factors diluting the strength of the effect might be the scanner noise, which may have unsystematically affected the task performance.

#### 4.2.2 Neurofunctional data

The present study showed an increased activation in the right cerebellum (Lobule VI) for the conjunction of both the non-phonological dyslexics in contrast to controls and the phonological dyslexics in contrast to controls. In light of the cerebellar deficit hypothesis, an effect in the cerebellum seems plausible. Nicolson et al. (2001) proposed that language-related regions of the cerebellum are affected in dyslexia. These regions are considered to be Lobule VI (e.g. Stoodley and Schmahmann, 2009) and VIIB in the neocerebellum. The cerebellar deficit hypothesis claims that a disruption of these areas leads to articulatory problems and subsequently to difficulties in phonological awareness to produce dyslexia. The language related regions of the cerebellum are physically distant from cerebellar sensorimotor (Lobule VIII) and association areas (Lobule II, Crus I and Crus II) (e.g. Schmahmann, 2010), but the present study did not explicitly examine automatization or motor deficits

In anatomical imaging studies, the cerebellum seems to be one of the locations where structural differences between dyslexics and controls are most consistently found (Pernet et al., 2009a,b). Anatomical findings have pointed out the involvement of the cerebellum in dyslexia by showing a smaller right cerebellar anterior lobe both in adults (Leonard et al., 2001) and in children (Eckert et al., 2003) with dyslexia. Similarly, the findings are in line with the idea that worse phonological performance in dyslexia is related to functional abnormalities in the cerebellum. A manual volume measurement of the right anterior cerebellar lobe which differentiates dyslexics and controls showed that this region is correlated with phonological processing in rapid naming and phoneme elision (Eckert et al., 2003). The bigger the gray matter volume of the right cerebellum, the greater the phonological abilities. Rae et al. (2001) found dyslexics to have greater cerebellar asymmetry than controls (right gray matter > left gray matter). In regard to phonological processing, the degree of cerebellar symmetry was correlated with the severity of dyslexics' phonological decoding deficit. The present study puts these findings into a neurofunctional perspective. We found an association between phonological ability, reading ability and activation intensity in the right cerebellum. Children with good phonological awareness and reading skills showed weaker activation in this region than children with poor phonological awareness and dyslexia. Thus, this correlation was found for the whole sample and not only for the dyslexic sample. Taking the behavioral and neurofunctional data together allowed a more detailed look into the neurobiological basis of dyslexia, and the behavioral results support the neurofunctional findings. A theoretical consolidation of the findings could posit a compensatory overactivation of a structurally underdeveloped region. Further research is needed to prove this hypothesis. To summarize, independently of the underlying cognitive deficit, activation differences in the right cerebellum seem to be strongly pronounced between dyslexics and controls on a phonological task.

### 4.3 Brain areas differentiating between dyslexic subgroups

Most importantly, the present study revealed not only differences between dyslexic children and controls in general, but also between the two dyslexia subgroups. The phonological dyslexics revealed stronger activation in the left SMA than the non-phonological dyslexics. This area is associated with verbal short term memory and is closely linked to a phonological rehearsal mechanism (Crottaz-Herbette et al., 2004; Davachi et al., 2001). One might therefore suggest that the phonological task employed in the present study induces recruitment of a phonological rehearsal mechanism in response to pseudowords in the group of phonological dyslexics, but this activation of phonological rehearsal was absent in the non-phonological dyslexic group. The correlation analysis showed that the faster the reaction time in the phonological task, the greater the activation intensity in the SMA. The SMA is active in a close time relation with movement execution even when the movement is simple (Brinkmann and Porter, 1979; Chen et al., 1991; Tanji, 1994), such as keystroke in the present paradigm. But there were no differences in reaction time data between the groups, so motion planning could not explain the SMA overactivation in phonological dyslexics alone.

Beyond that, phonological dyslexics showed greater activation than non-phonological dyslexics in the inferior frontal gyrus (area 44). In a more detailed look at the function of this area, there is accumulating evidence that the posterior aspect of the left IFG, and in particular area 44, is closely involved in fine-grained phonetic analysis such as segmentation (Zatorre et al., 1992, 1996). Neuroimaging studies with healthy participants have demonstrated that the inferior frontal gyrus shows increased activation during phonological tasks such as segmentation, discrimination and monitoring (for a review see Burton, 2001; Burton et al., 2000), but not during passive listening or sensory conditions. In the present study, a phonological task was difficult for the phonological dyslexics and may have resulted in hyperactivation in area 44 in contrast to the non-phonological dyslexics, who did not have difficulties on this task. With regard to the function of area 44, there is an ongoing debate about the neurofunctional differences in phonological processing between dyslexic and normally reading children. Some studies have found that dyslexic children showed deactivation in the left IFG (Cao et al., 2006; Georgiewa et al., 1999), whereas other studies have not found any left-hemisphere differences (Rumsey et al., 1997; Temple et al., 2001). Most phonology-based studies have used different tasks to evaluate the effects of phonological processing on brain activation, and these varied tasks may lead to distinct activation patterns. Moreover, most studies have used visual stimuli to tap into phonological processing, which may involve additional grapheme-to-phoneme conversion processes that interact with actual phonological processing. The present study used auditory stimuli to measure real phonological processes instead of grapheme-to-phoneme conversion. Phonological tasks with auditory stimuli activate rather posterior and superior parts of the IFG, whereas the processing of visual stimuli predominantly involves anterior and inferior parts of the IFG (Burton, 2001).

The present study also found increased activation for phonological dyslexics compared to non-phonological dyslexics in the right anterior dorsal insula, whereas better phonological awareness corresponded to lower activation intensity in this area. In previous lesion studies (e.g. Flynn, 1999), an involvement of the insula in language processing has often been reported. Although the insula is involved in many aphasic syndromes (Kreisler et al., 2000), its exact role is still being discussed. Functional imaging has studies that have reported the involvement of the anterior dorsal part of the left and right insula in language processing tasks (e.g. lexical decision making or semantic judgments) and in phonological processing (for a review see Bamiou et al., 2003), whereas the specialization within the insular cortex is still under discussion. Our results are a perfect complement to these findings, showing the involvement of the right insula in a phonological discrimination task distinguishing between dyslexic subgroups.

Beyond that, the non-phonological dyslexics exhibited increased activation in the left supramarginal (PFcm) and angular gyri (PGp) in comparison to the phonological dyslexics. The supramarginal gyrus is related to phonological processing (Gelfand and Bookheimer, 2003; Geschwind, 1970; Petersen et al., 1988; Price, 2000; Vigneau et al., 2006; Zatorre et al., 1992) and is crucial in phonological short-term memory tasks (Henson et al., 2000; Paulesu et al., 1996) in healthy participants, whereas the angular gyrus is more involved in the understanding of written and spoken language (Geschwind, 1965; Hart and Gordon, 1990). The arcuate fasciculus (Catani et al., 2005; Frey et al., 2008; Parker et al., 2005; Saur et al., 2008) is the main fiber pathway connecting Broca's area (area 44/45) with all inferior parietal regions. Caspers et al. (2011) showed that PGa and PGp have the highest densities of fiber tract connections to Broca's area via this ventral route, whereas PGp showed consistent connection patterns with auditory areas. This relation represents the key role of the inferior parietal lobe in auditory processing, which is supported by several neurofunctional studies (Price, 2000; Saur et al., 2008). The non-phonological dyslexics had no problems in the processing of a phonological awareness task (BAKO T-value > 45) but they still showed neurofunctional differences in an area involved in phonological processing. As non-phonological dyslexics showed specific activation profiles in this area which were moderated in phonological dyslexics, one could assume that they did not struggle with phonological segmentation but rather with phonological storage. Because of the strong connection between the inferior parietal lobe and the inferior frontal gyrus, a dysfunction in the inferior parietal lobe could have an impact on the mode of operation in the connected area. These findings are in line with the offline data from the BAKO test of phonological awareness, which indicated that the non-phonological dyslexics achieved higher test scores than the phonological dyslexics but nevertheless performed below the level of the controls.

Thus, the two dyslexia subgroups investigated in this study had patterns of brain activation which were distinct from each other in a direct comparison. These patterns of brain activation seem to be related to behavioral patterns, which may provide an indication of different phonological processing strategies and serve as the basis for meaningful interpretation. These different strategies were not visible on the behavioral level, because the percentages of correct answers in the phonological task did not differ significantly between the dyslexic subgroups.^5^

## 5 Conclusion

The present paper has shown that, despite underlying cognitive deficits and in contrast to controls, dyslexic children showed increased activation in right cerebellum while performing a phonological task. Additionally, differences among cognitive subtypes of dyslexic children with and without a phonological deficit were demonstrated. The present study thus contributes to resolving the problem of heterogeneity in neuroimaging results of dyslexia, demonstrating that differential activation profiles between dyslexic and normal readers may be found depending on the cognitive deficit pattern underlying the children's reading difficulties. Further research is needed to differentiate between several cognitive subtypes of dyslexia on the basis of neuroimaging studies.

## Figures and Tables

**Table A.1:** Neuroimaging results of the phonological network for the total sample, controls, phonological dyslexics, non-phonological dyslexics separately.

Cluster size (voxels)	Local maximum in macroanatomical structure	x	y	z	Tmax	Percent of cluster volume in cytoarchitectonic area	
*Ptask*–*Ctask total sample*							
3018	Left precentral gyrus (cluster extends into left inferior frontal gyrus)	− 58	4	22	6.26	47.3	Area 44
						2.8	Area 6
						8.8	Area 45
						2.9	Area 4a
						3.0	Area 4p
						2.1	Area 3a
						1.6	Area 3b
2630	Left SMA	− 4	16	46	11.45	13.2	Area 6
						5.5	Area 6
816	Left lingual gyrus	− 14	− 70	2	5.73	13.7	Area 17
						9.1	Area 18
						2.7	Area 18
						1.2	Area 17
						3.6	hOC3v (V3v)
						1.1	hOC4v (V4)
690	Right inferior frontal gyrus	36	24	− 8	6.21		
293	Right cerebellum	34	− 66	− 28	5.21		Lobule VIIa Crus I (Hem)
							Lobule VI (Hem)
291	Left thalamus	− 10	− 6	4	4.80	33.6	Th-Prefrontal
						1.6	Th-Premotor
						2.4	Th-Temporal
						1.4	Th-Motor
80	Left superior parietal lobule	− 28	− 60	46	4.58	18.1	hIP3

*Con Ptask*–*Ctask*							
693	Left SMA	− 2	16	48	5.7	3.2	Area 6
180	Left inferior frontal gyrus	− 48	6	18	4.40	9.1	Area 44
165	Left insula	− 36	22	− 2	4.30		
73	Left precentral gyrus	− 42	− 4	42	4.25		
11	Left superior frontal gyrus	− 24	− 4	58	3.45		

*PhoDys Ptask*–*Ctask*							
2343	Left SMA	− 4	16	46	9.80	10.0	Area 6
1981	Left precentral gyrus (cluster extends into left inferior frontal gyrus)	− 58	4	22	6.84	41.3	Area 44
						1.0	Area 6
						3.9	Area 3b
						2.6	Area 3a
						1.6	Area 4p
753	Right cerebellum	42	− 62	− 32	5.76	22.1	Lobule VI (Hem)
						8.2	Lobule VIIa Crus I (Hem)
560	Right inferior frontal gyrus	34	26	− 6	6.34		
191	Left calcarine sulcus	4	− 88	8	4.40	3.4	Area 17
						3.2	Area 18
						2.2	Area 17
189	Left lingual gyrus	− 14	− 72	0	5.09	3.7	Area 17
						4.4	Area 18
						2.8	hOC3v (V3v)
						1.0	hOC4v (V4)
174	Left thalamus	− 14	− 16	6	4.66	22.0	Th-Prefrontal
						25.3	Th-Premotor
89	Left postcentral gyrus	− 52	− 8	44	4.63	1.0	Area 6
						2.0	Area 4a

*NonPhoDys Ptask*–*Ctask*							
767	Left SMA	− 6	10	58	5.76	6.9	Area 6
351	Left insula	− 34	14	12	5.63		
195	Right cerebellum	38	− 68	− 28	4.32	3.3	Lobule VIIa Crus I (Hem)
						4.6	Lobule VI (Hem)
144	Left lingual gyrus	− 14	− 72	0	4.21	2.4	Area 17
						2.4	Area 18
						2.0	hOC4v (V4)
						2.1	hOC3v (V3v)
87	Left cuneus	− 8	− 84	14	3.86	2.4	Area 17
						1.7	Area 18
73	Left temporal pole	− 44	8	− 18	4.51		Area 10
25	Left postcentral gyrus	− 52	− 6	44	3.97		
24	Right middle cingulate cortex	10	26	36	3.76		

Notes: Extent k ≥ 10 voxel (all local maxima significant at p < .001uncorrected). The table shows cluster-wise the number of voxels in the cluster, the macro-anatomical structure and the MNI coordinates of the local maximum, the maximum T value at the local maximum and the cytoarchitectonically defined location of the local maximum assessed with the SPM Anatomy Toolbox (Eickhoff et al., 2005).

**Table A.2:** Effect of group on brain activation. Single contrast of phonological dyslexics versus controls (PhoDys > Con) and non-phonological dyslexics versus controls (NonPhoDys > Con) (p-value = .001; uncorrected; threshold = 10).

Cluster size (voxels)	Local maximum in macroanatomical structure	x	y	z	T_max_	Percent of cluster volume in cytoarchitectonic area	
*PhoDys* > *Con*							
231	Right cerebellum	10	− 86	− 18	3.95	6.9	hOC3v (V3v)
						1.4	Lobule VIIa Crus I (Hem)
						1.4	Area 18
						1.1	Lobule VI (Hem)
						1.9	hOC4v (V4)
74	Right cerebellum	18	− 54	− 28	3.64	3.7	Lobule VI (Hem)
38	Right inferior temporal gyrus	40	− 64	− 8	3.55	2.1	Hipp (CA)
18	Right hippocampus	36	− 24	− 14	3.51	1.3	Hipp (FD)
17	Left precentral gyrus	− 34	8	32	3.53		
15	Right cerebellum	42	− 62	− 32	3.56		
14	Left precentral gyrus (cluster extents into left inferior frontal gyrus)	− 60	6	20	3.69	1.0	Area 44

*NonPhoDys* > *Con*							
119	Right hippocampus	30	− 34	− 6	4.24	8.4	Hipp (CA)
						25.5	Hipp (FD)
92	Right middle occipital gyrus	38	− 68	12	4.23		
88	Right cerebellum	24	− 52	− 32	4.18	3.4	Lobule VI (Hem)
35	Right frontal pole	2	60	− 8	3.70		
26	Right insula	40	− 6	− 4	3.64		
26	Left lingual gyrus	− 18	− 76	− 2	3.32	1.4	hOC4v (V4)
18	Left middle frontal gyrus	− 28	18	42	3.68		

**Table 1:** Performance of phonological dyslexics (PhoDys), non-phonological dyslexics (NonPhoDys) and controls (Con) in the initial study.

	PhoDys (N = 17)	NonPhoDys (N = 14)	Con (N = 13)
Age mean (range)	9.9 (range: 8.8–11.2)	9.8 (range: 8.7–10.11)	9.6 (range: 9.0–10.5)
Sex	8 ♀	8 ♀	5 ♀
	9 ♂	6 ♂	8 ♂
First language	Monolingual German	Monolingual German	Monolingual German
Handedness	Right (1 left)	Right	Right
Grade German	C (10); D (6); ns (1)	B (1); C (7); D (2); ns (4)	A (1); B (9); C (2); ns (1)
Familial risk	9	5	0
SLS Mean ± SD	74.88 ± 7.7	74.57 ± 12.7	118.69 ± 15.9
IQ mean (range)	106.35 (85–145)	109.35 (89–133)	122.61 (105–147)
BAKO Mean ± SD	33.06 ± 5.2	45.71 ± 4.87	55.92 ± 11.21
KiTAP visual scanning	53.6 ± 8.6	49.4 ± 7.9	57.5 ± 11.8

Notes: Grade *German* contains to the last school certificate (A = “sehr gut”; B = “gut”; C = “befriedigend”; D = “ausreichend”; ns = no specified in case history). Familial risk describes the number of children when at least one of the parent reported to have dyslexia in case history. The reading screening (SLS) contains the reading quotient which is scaled like the intelligence quotient ± the standard deviation (SD). The IQ refers to the age norms ± SD, whereas the results of the phonological awareness test (BAKO) bears upon the total t-values. Visual scanning (KiTAP) was performed as an additional psychometric assessment and refers to t-value ± the standard deviation.

**Table 2:** Performance of phonological dyslexics (PhoDys), non-phonological dyslexics (NonPhoDys) and controls (Con).

Group	N	Ctask RT (ms) ± SD	Ptask RT (ms) ± SD	Ctask %-correct ± SD	Ptask %-correct ± SD
*IQ total sample*					
PhoDys	17	1200 ± 300	1730 ± 400	88.3 ± 5.5	64.9 ± 15.3
NonPhoDys	14	1190 ± 400	1920 ± 400	81.9 ± 15.1	76.8 ± 14.4
Con	13	1090 ± 200	1600 ± 400	90.8 ± 3.8	72.1 ± 17.3
Total	44	1106 ± 300	1750 ± 400	87.08 ± 9.9	70.89 ± 16.1

*IQ*-*matched sample*					
PhoDys	15	1210 ± 300	1720 ± 400	88.56 ± 5.8	64.58 ± 16.3
NonPhoDys	12	1090 ± 100	1880 ± 300	81.64 ± 16.1	77.35 ± 15.6
Con	7	1150 ± 300	1600 ± 400	91.03 ± 3.7	73.90 ± 14.8
Total	34	1150 ± 200	1750 ± 400	86.68 ± 10.9	71.01 ± 16.4

Notes: The table describes the mean of reaction time (RT) per milliseconds and the percentages of correct answers (%-correct) for the control task (Ctask) and phonological task (Ptask) with the standard deviation (SD).

**Table 3:** Neuroimaging results of the conjunction analysis of phonological dyslexics (PhoDys) versus controls (Con) and non-phonological dyslexics (NonPhoDys) in contrast to Con.

Cluster size (voxels)	Local maximum in macroanatomical structure	x	y	z	T_max_	Percent of cluster volume in cytoarchitectonic area	
*Conjunction PhoDys > Con ∩ NonPhoDys > Con*							
13	Right cerebellum	20	− 54	− 28	3.57	87.9	Lobule VI (Hem)

Notes: Notes: Extend k ≥ 10 voxel (all local maxima significant at p < .001uncorrected). The table shows cluster-wise the number of voxels in the cluster, the macro-anatomical structure and the MNI coordinates of the local maximum, the maximum T value at the local maximum and the cytoarchitectonically defined location of the local maximum assessed with the SPM Anatomy Toolbox (Eickhoff et al., 2005).

**Table 4:** Neuroimaging results of the direct comparison of dyslexic children with (PhoDys) and without phonological deficit (NonPhoDys).

Cluster size (voxels)	Local maximum in macroanatomical structure	x	y	z	T_max_	Percent of cluster volume in cytoarchitectonic area	
*PhoDys* > *NonPhoDys masked incl*. *PhoDys* > *Con*							
207	Left SMA	0	14	48	4.72	1.4	Area 6
52	Left precentral gyrus (cluster extends into left inferior frontal gyrus)	− 40	8	32	3.68	0.8	Area 44
40	Right insula lobe	32	24	− 4	4.08		
24	Left precentral gyrus (cluster extends into left inferior frontal gyrus)	− 56	4	22	3.89	1.2	Area 44

*NonPhoDys* > *PhoDys masked incl*. *NonPhoDys* > *Con*							
28	Left supramarginal gyrus	− 48	− 48	28	4.32	5.0	IPC (PFcm)
13	Left angular gyrus	− 46	− 60	24	3.42	0.4	IPC (PGp)

Notes: Extend k ≥ 10 voxel (all local maxima significant at p < .001uncorrected).

## Footnotes

1 Normalization of mean-EPI on the EPI template for each participant because not all children stayed in scanner until the end of the anatomical scan, which was last in each session. Localization of activation is as precise as the resolution of the EPI template.

2 To exclude the possibility that the results of the behavioral were due to the level of intelligence, the same analysis of the behavioral data was carried out with a sub-sample matched for IQ. The results were comparable to those of the analysis involving all subjects.

3 In order to test normal distribution of the test variables in each sub-group, Kolmogorov–Smirnov tests were run separately for PhoDys, NonPhoDys, and Controls. All tests were far from significant, indicating normal distribution even within these sub-samples. PhoDys: BAKO: p = .496; SLS: p = .601; IQ: p = .607; accuracy Ptask: p = .578; RT Ptask: p = .962; accuracy Ctask: p = .486; RT Ctask: p = .144; and KiTAP (scanning): p = .836. NonPhoDys: BAKO: p = .687; SLS: p = .707; IQ: p = .992; accuracy Ptask: p = .414; RT Ptask: p = .882; accuracy Ctask: p = .273; RT Ctask: p = .882; and KiTAP (scanning): p = .720. Controls: BAKO: p = .859; SLS: p = .982; IQ: p = .767; accuracy Ptask: p = .554; RT Ptask: p = .764; accuracy Ctask: p = .490; RT Ctask: p = .845; and KiTAP (scanning): p = .955. However, given the small sample sizes of each group, it cannot be totally excluded that the Kolmogorov–Smirnov test might not have been sensitive enough to detect violations of the normal distributions.

4 In order to assess whether activation in brain regions distinguishing dyslexia subtypes also shows effects of phonological proficiency in normal readers, we ran an additional analysis. This analysis included the beta estimate differences for the control group at the local maxima identified in the total sample. None of these effects were even close to significance (all p > .21). Whether this is due to the relatively small sample size of controls (n = 13) or a ceiling effect cannot be clarified with the present data set alone.

5 Note that, depending on the empirical situation, categorical (i.e. subjects with vs. without phonological problems) vs. continuous approaches (i.e. using phonological awareness as a continuous covariate) might be chosen. These two approaches yield similar results (Heim et al., 2010), but the one or the other might be more appropriate under different circumstances. If no subgroups can be objectively distinguished from one another, and yet the impact of a cognitive dimension on the fMRI signal is under investigation, a continuous approach is preferable. If, however, there is a clear, objective criterion for clinically impaired vs. normal performance, such as a T value of 40 as was used in the present study, a categorical approach might be more informative. This is in particular the case if previous data have demonstrated a taxonomy of distinct subgroups, such as e.g. Valdois et al. (2003) or Heim et al. (2008).
